# Integrative Multi-Omics Analysis Reveals Nutritional Metabolite Diversity and Regulatory Mechanisms in *Ocimum basilicum*

**DOI:** 10.3390/life16060890

**Published:** 2026-05-26

**Authors:** Yuanyuan Zhang, Manman Xu, Zizuo Miseme, Shiqi Yang, Xiangrong Chen, Cong Zhao, Yujian Wang, Jingtian Yang

**Affiliations:** 1 College of Biology and Pharmacy, Mianyang Teachers’ College, Mianyang 621000, China; 2 Key Laboratory for Quality Control and Evaluation of Traditional Chinese Medicine in Mianyang, Mianyang Teachers’ College, Mianyang 621000, China; 3 Forest Ecology and Conservation in the Upper Reaches of the Yangtze River Key Laboratory of Sichuan Province, Engineering Research Center for Forest and Grassland Disaster Prevention and Reduction at Mianyang Teachers’ College of Sichuan Provincial Department of Education, School of Life Sciences, Mianyang Teachers’ College, Mianyang 621000, China; 4 School of Environmental Science and Engineering, Southwest Jiaotong University, Chengdu 611756, China; 5 School of Geography and Environment, Mianyang Teachers’ College, Mianyang 621000, China

**Keywords:** *Ocimum basilicum*, multi-omics, nutritional metabolites, metabolomics, transcriptomics, regulatory mechanisms

## Abstract

*Ocimum basilicum* is widely used as both a culinary and medicinal plant; however, its nutritional metabolite composition, functional relevance, and underlying regulatory mechanisms remain incompletely characterized. To systematically profile nutritional metabolites and explore their potential biological relevance in *O. basilicum*, an integrative multi-omics strategy that combined UPLC-MS/MS-based metabolomics, transcriptomics, network pharmacology, and molecular docking was deployed herein. A total of 443 nutritional metabolites across four accessions were identified, including vitamins, saccharides, amino acids and lipids. Of these, Vitamin A1 (retinol) and N-acetyl-L-tryptophan were found to be important metabolites that could have functional significance. Predictive network pharmacology and molecular docking analyses suggested potential in silico interactions between these metabolites and disease-associated targets, including ESR2 and MAPK1; these findings await experimental validation. Transcriptomic analysis also showed that genes involved in Vitamin A1 biosynthesis (PSY, LCYB) were expressed, and the expression patterns of the genes were validated by the qPCR analysis, in which expression level was largely consistent with the transcriptome results. Specifically, the accessions G083 and G082 showed high values of Vitamin A1 and N-acetyl-L-tryptophan, respectively, suggesting that they may also be interesting germplasm for functional food innovation and the development of nutraceuticals. Overall, this study offers a comprehensive multi-omics dataset and mechanistic insights that can help in the targeted use of *O. basilicum* for nutritional and functional applications.

## 1. Introduction

*Ocimum basilicum*, an annual herbaceous species of the Lamiaceae family, is widely cultivated and extensively utilized worldwide as a medicinal and edible plant [1,2]. It serves as a vital component of traditional medicine [3]. Characterized by its pungent-sweet flavor and warming properties, *O. basilicum* is known to alleviate wind-induced symptoms, resolve dampness, harmonize the digestive system, regulate qi (a core concept in traditional Chinese medicine referring to the body’s vital functional energy and physiological balance), invigorate blood circulation, detoxify, and reduce swelling. This herb is employed in the treatment of various ailments, including headaches caused by colds, feverish coughs, heatstroke, and indigestion [4,5]. Contemporary research attributes these therapeutic benefits to its bioactive constituents [6]. Most existing studies have focused predominantly on the volatile oils and essential oil components of *O. basilicum* [7], with limited attention to its non-volatile nutritional metabolites. Moreover, the regulatory mechanisms governing the biosynthesis of these nutritional compounds remain largely unexplored. *O. basilicum* contains volatile oils [7], flavonoids [8], and coumarins [9], which possess diverse pharmacological activities, including anti-inflammatory and anti-anxiety ones, as well as sedative, hypoglycemic, constipation-relieving, and anti-ulcer activities [10].

*O. basilicum*’s unique and strong flavor is essential to many dishes [11]. *O. basilicum* is not only used to add flavour but also is highly nutritional, since it contains an abundance of nutrients such as vitamins, amino acids, carbohydrates, and lipids [12,13]. All of these components play a vital role in plant growth and development [14]. Most importantly, their synergistic interactions impart a myriad of health benefits to humans [15]. In combination, these properties make *O. basilicum* a valuable plant for culinary use, as well as for potential therapeutic applications.

From the plant’s point of view, nutrients function synergistically in its physiological processes [10]. Vitamins act as antioxidants that bind and neutralize free oxygen radicals produced in the metabolic process. This action results in protection against cellular oxidative damage and allows the normal functioning of cells [16]. Amino acids are the basic component of proteins, and are crucial in several physiological activities such as photosynthesis, respiration and the transportation of substances [17]. Carbon is the main energy source for plants, and it also helps in cell wall formation, which gives structure to the plant [18]. Lipid metabolites such as fatty acids and phospholipids are essential for the fluidity and stability of biological membranes [19]. Besides, they are also involved in plant hormone synthesis and signal transduction. For example, under stress conditions, antioxidant vitamins C and E have been found to act synergistically to increase the antioxidant capacity of plants [20]. Amino acids regulate the metabolism of nitrogen, which provides the plant with more energy and material resources so that it can better withstand unfavorable conditions [21]. Under stress, the metabolism and distribution of carbohydrates is modified in accordance with the plants’ energy needs [22]. Lipid metabolites assist plants in acclimating to challenging environments through the dynamic regulation of biomembrane architecture and physiological activity, as well as by participating in the synthesis and signaling of plant hormones [23].

The nutrients in *O. basilicum* interact synergistically to promote human health [10]. Vitamins and amino acids collaborate in metabolic processes: vitamins serve as coenzymes or cofactors in enzymatic reactions, facilitating amino acid metabolism and utilization, while amino acids provide precursors for vitamin synthesis (e.g., tryptophan for niacin) [24]. Carbohydrates and lipid metabolites supply energy, with unsaturated fatty acids regulating blood lipid levels and reducing cardiovascular risk. Moreover, they work in conjunction with dietary fiber to promote intestinal homeostasis and maintain glycemic equilibrium [25,26]. For instance, vitamin E, linoleic acid, and dietary fiber jointly contribute to cardiovascular disease prevention. Vitamin E protects vascular endothelial cells, linoleic acid optimizes cholesterol levels to reduce the risk of atherosclerosis, and dietary fiber decreases cholesterol absorption and promotes its excretion, further lowering blood lipid levels [27].

While volatile constituents have historically been the primary focus of investigations regarding *O. basilicum* [28,29,30,31], a comprehensive elucidation addressing the explicit types, precise quantities, and underlying functional mechanisms of its nutritional components remains incomplete. To fill this knowledge gap, an integrated multi-platform workflow was implemented, seamlessly weaving together metabolomics (UPLC-MS/MS), transcriptomics, network pharmacology, and molecular docking. We want to fully investigate the nutrient profile of *O. basilicum*, elucidate its medicinal potential and study the metabolic pathways of major metabolites. Metabolomics will enable us to precisely quantify the vitamins, amino acids, carbohydrates and lipids contained in *O. basilicum* and thus provide a solid data set on its nutritional value. Using network pharmacology and molecular docking, we aim to build and study a network model of metabolite–target–disease–pathway. The medicinal potential and mechanism of these key metabolites will be revealed from this analysis, and insights related to the pharmacological application of these metabolites will be provided. Transcriptomics will, in addition, identify the main genes involved in the production of these metabolites, thereby adding to the knowledge on the metabolic regulation of *O. basilicum*. The theoretical and practical significance of this study is great. Theoretically, it enhances our understanding of *O. basilicum*’s dual nutritional and medicinal value and offers innovative research strategies and methodologies. The results will give scientific data for the development and use of *O. basilicum*, on the practical level. They will advocate for its use in food, pharmaceutical and health product industries, boost development of the *O. basilicum* industry and be a good reference for other medicinal-edible plants.

## 2. Materials and Methods

### 2.1. Plant Materials

Four *Ocimum basilicum* accessions (G002, G083, G082 and G122) were collected from the Nanyao Germplasm Resources Nursery of the Institute of Tropical Crop Variety Resources, Chinese Academy of Tropical Agricultural Sciences, Danzhou City, Hainan Province. Nine healthy and uniform plants of each accession without pests and diseases were selected and then were divided into three biological replications, which were three plants each. The leaves of each plant were collected (10 g each), immediately frozen with liquid nitrogen and kept at −80 °C for further analysis.

### 2.2. Sample Preparation

For sample preparation, dehydration of the fresh basil leaves was executed via a vacuum freeze-dryer (Scientz-100F, Ningbo Scientz Biotechnology Co., Ltd., Ningbo, China) over a continuous duration of 63 h. Following this desiccation process, the resulting material was pulverized into a homogeneous fine powder using a grinding mill (MM400, Retsch GmbH, Haan, Germany; 30 Hz, 90 s), following the protocol described previously [32]. Sample aliquots (50 mg) were measured on an MS105DM balance and extracted in 1200 µL of pre-cooled (−20 °C) 70% methanol with an internal standard. The samples underwent six rounds of vortexing (30 s each, every 30 min). Following centrifugation (12,000 rpm, 3 min), the extracts were filtered through a 0.22 µm membrane into vials for UPLC-MS/MS evaluation.

### 2.3. HPLC Conditions

Liquid chromatography separation was conducted on an Agilent SB-C18 column (1.8 µm, 2.1 × 100 mm; Agilent Technologies, Santa Clara, CA, USA). The binary mobile phase utilized water and acetonitrile as solvents A and B, respectively, with both acidified by 0.1% formic acid. The mobile phase B gradient was applied as follows: 5% at 0.00 min, linearly increasing to 95% by 9.00 min, held at 95% until 10.00 min, decreased to 5% at 11.10 min, and maintained at 5% until 14.00 min for column equilibration. The system operated at 40 °C with a flow rate of 0.35 mL/min and a 2 µL injection volume.

### 2.4. Mass Spectrometry Conditions

Mass spectrometry was done using an ESI source at 500 °C and an ion spray voltage of 5500 V (positive) and −4500 V (negative). The gas pressures were chosen to be 50 psi (Gas I), 60 psi (Gas II) and 25 psi (curtain gas) with a high-collision gas setting. Scans were carried out in single-reaction monitoring (MRM) mode with medium-pressure nitrogen. The declustering potential and collision energy were optimized for each MRM transition and monitored specific ion pairs for each of their elution windows.

### 2.5. Metabolite Identification and Relative Quantification

The compound annotation was based on our in-house database, with the different peak areas (counts per second, CPS) measured by triple quadrupole (MRM) chromatograms representing the relative abundances of the various metabolites. Peak integration and correction were handled by the MultiQuant software 3.0.3. To identify differentially accumulated metabolites, we applied a combination of PCA, OPLS-DA, fold-change (FC), and Student’s *t*-tests. Specifically, compounds with a VIP ≥ 1 (from the OPLS-DA model, *n* ≥ 3) and |log2FC| ≥ 1.0 were deemed significant. Finally, these key metabolites were mapped to biological pathways using the KEGG Compound and Pathway databases (http://www.kegg.jp/).

### 2.6. Total RNA Extraction

The isolation of total RNA from the botanical tissues was executed via ethanol precipitation paired with a CTAB-PBIOZOL extraction framework. The final RNA pellet was dissolved in 50 µL of DEPC-treated water, and its concentration and integrity were subsequently assessed using a Qubit fluorometer (Thermo Fisher Scientific, Waltham, MA, USA) and a Qsep400 high-throughput fragment analyzer (BiOptic Inc., New Taipei City, Taiwan).

### 2.7. Library Construction and Quality Control

The preparation of cDNA libraries was executed in compliance with the standard strand-specific library preparation protocol. In brief, the capture of poly(A) mRNA from the total RNA pool was mediated by Oligo(dT) magnetic beads, and the poly(A) mRNA was subsequently subjected to thermal shearing into short segments utilizing a fragmentation buffer under elevated temperatures. Random hexamer primers were used to make the first strand of cDNA from these templates. In the second strand, dTTP was replaced with dUTP to preserve the directionality of the strands [33]. The double-stranded cDNA was then end-repaired, A-tailed and ligated with sequencing adapters. Size selection targeting fragments spanning 250 to 350 bp were performed via magnetic beads, followed by PCR amplification to yield the finalized sequencing library [34]. Library concentrations were measured using a Qubit fluorometer, and fragment size distributions were assessed with a Qsep400 analyzer.

### 2.8. Sequencing

Metware Biotechnology Co., Ltd. (Wuhan, China) provided Illumina sequencing services. Quality-verified libraries were combined based on data and concentration needs. These pooled samples were then sequenced to produce 150 bp paired-end reads. Mechanistically, the operational framework of this technology relies on the core principle of sequencing-by-synthesis. Within the microfluidic flow cell of the sequencer, molecular amplification is mediated by the introduction of adapter primers, DNA polymerase, and four types of fluorescently labeled dNTPs. As the complementary strand is elongated within each localized sequencing cluster, the specific integration of each fluorescently labeled dNTP liberates a corresponding fluorescent signal. These optical emissions are systematically captured by the sequencer, whereupon specialized computer software translates the data into distinct sequencing peaks, thereby resolving the primary nucleotide sequence of the target DNA fragment.

### 2.9. Pharmacological Functional Analysis

PubChem (https://pubchem.ncbi.nlm.nih.gov/) provided the structural data for our candidate metabolites. For target prediction, the Swiss Target Prediction database (http://www.swisstargetprediction.ch/) identified human proteins matching a >0.1 probability cutoff, which we subsequently mapped into an interaction network by utilizing Cytoscape 3.9.1.

The STRING 12.0 database (https://cn.string-db.org/) generated the initial Homo sapiens PPI network using a high confidence threshold of 0.9. Subsequently, the CytoHubba plugin in Cytoscape 3.9.1 calculated Maximal Clique Centrality (MCC) values to identify core nodes, allowing us to visualize an exclusive network of key targets.

The STRING 12.0 platform handled the GO functional annotation (categorized into biological processes, cellular components, and molecular functions) and KEGG enrichment (https://www.kegg.jp/) [35] for the prioritized targets. All subsequent data visualizations were carried out in the R 4.3.3 software.

Data regarding metabolite-associated diseases were acquired from PubChem (https://pubchem.ncbi.nlm.nih.gov/), while relevant disease targets (relevance score > 5) were identified through GeneCards (https://www.genecards.org/). To visualize the global interplay between these metabolites, targets, pathways, and diseases, an integrative network was generated using Cytoscape 3.9.1.

### 2.10. Molecular Docking

The 3D structures of the core proteins and candidate metabolites were obtained from UniProt (https://www.uniprot.org/) and PubChem (https://pubchem.ncbi.nlm.nih.gov/), respectively. Molecular docking was then performed using the cavity-detection tool in CB-DOCK2 (https://cadd.labshare.cn/cb-dock2/). Upon completing the binding orientation calculations, the resulting PDB spatial datasets mapping the complexed macromolecular receptors and small-molecule ligands were comprehensively rendered and visualized through the deployment of Discovery Studio 2019.

### 2.11. Expression Analysis of Candidate Genes

Total RNA (1.0 µg) was reverse-transcribed into cDNA using the TransScript^®^ All-in-One First-Strand cDNA Synthesis SuperMix for qPCR. The qPCR assays were performed on a CFX96™ Real-Time PCR System (Bio-Rad, Hercules, CA, USA) using PerfectStart^®^ Green qPCR SuperMix. The 20 µL reaction mixture contained 10.0 µL of SuperMix, 0.8 µL of PrimerMix, 1.0 µL of cDNA, and nuclease-free water. The thermal cycling conditions were 94 °C for 30 s, followed by 40 cycles of 94 °C for 5 s and 60 °C for 30 s. Technical triplicates were systematically executed for every targeted gene across all amplification reactions. The evaluation of the relative gene expression profiles was accomplished through the implementation of the standard 2^−ΔΔCt^ method. Oligonucleotide design for the gene-specific primers was performed by utilizing the Primer Premier 5.0 software framework (Table 1).

### 2.12. Data Analysis

Microsoft Office 2021 handled basic data statistics, while Origin 2021 generated the histograms. The multidimensional plots were created in R; pie charts, PCA, and Venn diagrams in v3.5.1; K-means and heatmaps in v4.2.0; and bubble charts in v4.2.2. Metabolite–gene network interactions were mapped using Cytoscape 3.9.1.

## 3. Results

### 3.1. Comprehensive Analysis of the Identified Nutritional Metabolites

**Figure 1:**
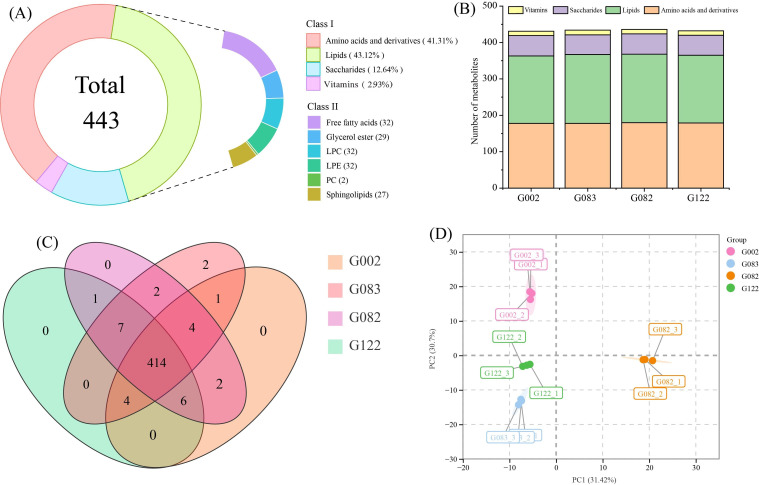
Comprehensive analysis of the nutritional metabolites. (**A**) Classification analysis of the nutritional metabolites. (**B**) The nutritional metabolites in each accession. (**C**) A Venn diagram of the nutritional metabolites. (**D**) Principal component analysis (PCA) based on the nutritional metabolites.

Across the four examined *Ocimum basilicum* accessions, a total of 443 distinct nutritional metabolites were successfully resolved (Figure 1A). Representing the predominant chemical categories, lipids as well as amino acids and their derivatives each constituted a proportion exceeding 40% of the entire metabolic profile. Additionally, saccharides accounted for 12.64% (56 individual compounds), while a relatively minor fraction of 2.93% was represented by vitamins. The detected lipid metabolites were further classified into six subcategories: Free fatty acids dominated this lipid profile, which also included glycerol esters, LPC, LPE, PC, and sphingolipids.

The analysis of individual materials revealed that the four accessions were similar in the number of nutritional metabolites identified, with G082 having the highest number of 436 and G002 the lowest at 431 (Figure 1B). A Venn diagram (Figure 1C) showed that 414 nutritional metabolites were shared by the four accessions. Two specific metabolites were found only in G083: cis-5-Dodecenoic acid and Vitamin A1.

PCA revealed that the first two principal components collectively accounted for 62.12% of the total variance (PC1, 31.42%; PC2, 30.70%) (Figure 1D). The close grouping of biological triplicates within the PCA space confirms the high reproducibility of our dataset. The four *O. basilicum* accessions also showed some differences in nutritional composition, each forming a distinct cluster. Notably, G082 was relatively distant from the other three accessions.

### 3.2. Comparative Profiling of the Differential Nutritional Metabolites

**Figure 2:**
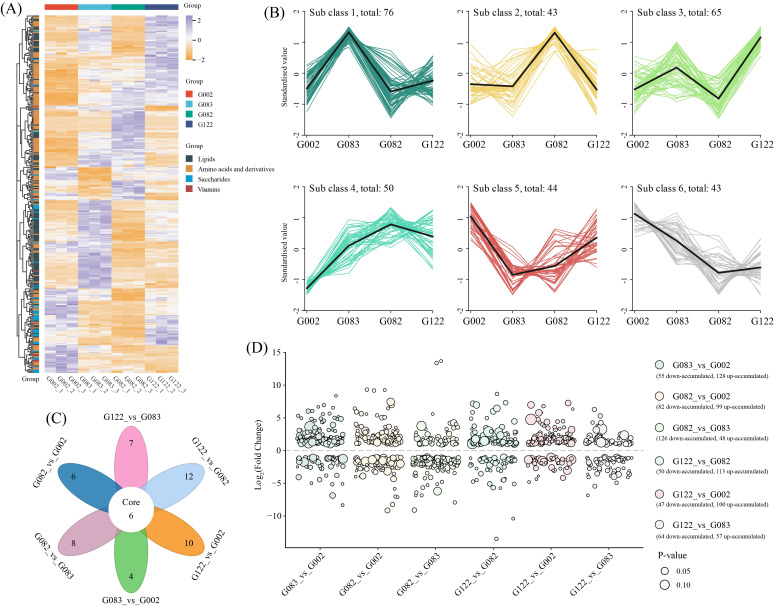
Analysis of the differential nutritional metabolites. (**A**) Heatmap of differential nutritional metabolites. (**B**) K-means analysis of differential nutritional metabolites. (**C**) A petal Venn diagram was constructed to illustrate the overlap of the differential nutritional metabolites. (**D**) Bubble plot of differential nutritional metabolites in each comparison group.

Using established differential metabolite screening criteria, we identified 321 differential nutritional metabolites (DNMs) and generated a heatmap (Figure 2A). These metabolites, which included 149 amino acids and derivatives, 118 lipids, 44 saccharides, and 10 vitamins, displayed distinct accumulation patterns across the *O. basilicum* accessions. A K-means clustering of these 321 DNMs partitioned them into six subclasses (Figure 2B). Subclass 1, the largest group, contained 76 DNMs that predominantly accumulated in G083. Subclass 2 consisted of 43 DNMs that highly accumulated in G082. Subclass 3 included 65 DNMs that were primarily abundant in G122, while subclass 4 had 50 metabolites that mostly accumulated in G002. Subclasses 5 and 6 contained 44 and 43 metabolites, respectively, which primarily accumulated in G002.

A Petal Venn diagram analysis of DNMs across comparison groups (Figure 2C) revealed six common metabolites: Glucopyranose 6-Hydroxydecanoate, D-Arabinose, 4-amino-5-oxo-5-(pentylamino) pentanoic acid, DMelezitose O-rhamnoside, N-Acetyl-L-Tryptophan, and Octanoyl arabinosylglucoside. Each comparison group also had unique DNMs. A bubble plot (Figure 2D) demonstrates variations in the number of DNMs among comparison groups. The G083_vs_G002 group exhibited the most DNMs (183 in total), with 128 up-accumulated and 55 down-accumulated in G083 compared to G002. The G082_vs_G002 group had 181 differential metabolites. The G122_vs_G083 group showed the fewest differential metabolites, with 57 up-accumulated and 64 down-accumulated in G122 relative to G083.

### 3.3. Predictive Profiling of Candidate Targets for Nutritional Metabolites

**Figure 3:**
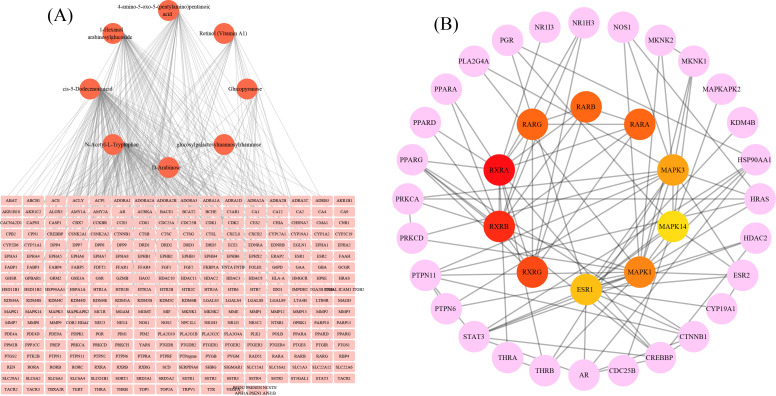
Analysis of putative targets for nutritional metabolites in *O. basilicum.* (**A**) Interaction network between putative targets and nutritional metabolites. (**B**) Protein–protein interaction (PPI) network of putative targets.

We identified eight metabolites, including two specific and six shared differential metabolites: 1-Hexanol arabinosylglucoside, 4-amino-5-oxo-5-(pentylamino) pentanoic acid, D-Arabinose, cis-5-Dodecenoic acid, Glucopyranose, glucosylgalactosylmannosylrhamnose, N-Acetyl-L-Tryptophan, and Retinol (Vitamin A1). Potential targets prediction revealed 284 associated targets, and we constructed a network regulation diagram to illustrate each candidate metabolite’s potential targets (Figure 3A).

The subsequent PPI network analysis of the predicted pool yielded 38 essential therapeutic targets (Figure 3B). Among these, the ten most central nodes included estrogen receptor 1 (ESR1) and three mitogen-activated protein kinases (MAPK1, MAPK3, MAPK14). Additionally, six retinoic acid receptor isoforms (RARA, RARB, RARG, RXRA, RXRB, and RXRG) were highly clustered at the network’s core, suggesting their pivotal biological roles.

### 3.4. Enrichment Analysis of Key Targets

**Figure 4:**
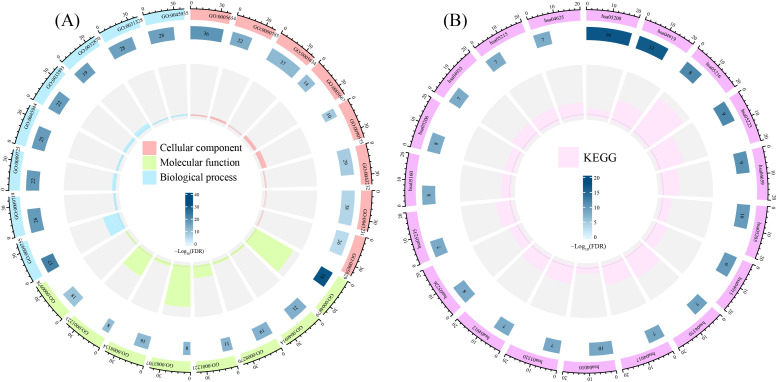
Enrichment profiling of core targets for nutritional metabolites. (**A**) Gene Ontology (GO) enrichment assessment. (**B**) Kyoto Encyclopedia of Genes and Genomes (KEGG) pathway enrichment evaluation. KEGG pathway data were obtained from the KEGG database (https://www.kegg.jp/) with permission from Kanehisa Laboratories [35].

Our GO pathway enrichment analysis of the 38 core targets reveals their significant involvement in a variety of biological processes, molecular functions, and cellular components (Figure 4A). The cellular component analysis highlighted 17 pathways, with a predominance in the nucleus and cytoplasm, indicating that most of these targets are nuclear or cytoplasmic proteins. The molecular function analysis identified 64 pathways, where these targets demonstrate transcription factor activity and enzyme binding capabilities. Furthermore, the biological process analysis pinpointed 525 pathways in which these targets are involved, including hormone-mediated signaling, responses to stimuli, and the regulation of gene expression and metabolism. These targets are implicated in various diseases, such as diabetes, cardiovascular diseases, and cancer, and are involved in key physiological processes like inflammation, immune responses, and cell proliferation, making them potential therapeutic targets. For example, ESR1 and ESR2 are linked to estrogen signaling and breast cancer development. Modulating these targets could provide therapeutic benefits for hormone-related cancers. Similarly, MAPK1, MAPK3, and MAPK14 are key regulators of the MAPK signaling pathway, which plays a role in cell proliferation, differentiation, and apoptosis, suggesting they could be useful for developing anti-cancer and anti-inflammatory therapies. In conclusion, the 38 key targets are involved in multiple biological processes and molecular functions, indicating their potential for use in treating various diseases. Further research into these targets could accelerate drug discovery and development.

The 38 core targets were significantly enriched across 135 distinct KEGG pathways (Figure 4B). These pathways are closely associated with various diseases and physiological processes, such as cancer, diabetes, cardiovascular diseases, and immune responses, indicating the targets’ potential for diverse pharmacological applications. For example, the “Pathways in cancer” and “PPAR signaling pathway” suggest that these targets could be used to develop anti-cancer and anti-inflammatory drugs. The “Estrogen signaling pathway” and “Thyroid hormone signaling pathway” indicate potential applications in hormonal regulation and the treatment of related diseases. Additionally, pathways like “Apoptosis” and “FOXO signaling pathway” are linked to cell survival and oxidative stress responses, which may be relevant for neurodegenerative and other diseases. Overall, these enriched pathways highlight the therapeutic potential of the targets across multiple disease areas. Further research into these pathways could deepen our understanding of the targets’ roles and accelerate the development of new drugs.

### 3.5. Combined Analysis of Functional Nutritional Metabolites, Targets, Pathways, and Efficacy

**Figure 5:**
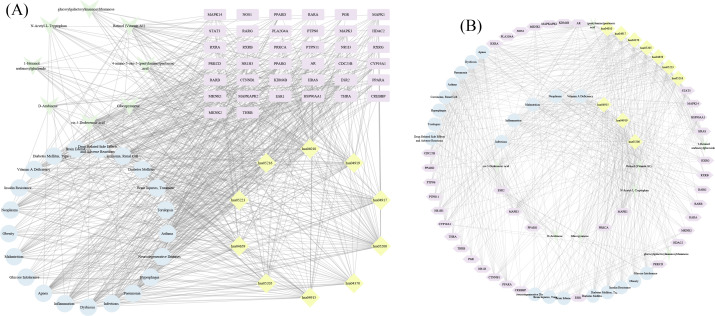
(**A**) Network diagram of functional nutritional metabolites–target–pathway–efficacy relationships. (**B**) Network diagram of core functional nutritional metabolites, targets, pathways and efficacy.

We analyzed eight candidate metabolites with potential pharmacological activity and constructed a network diagram (Figure 5A) integrating eight core functional nutritional metabolites, 21 diseases, 38 key targets, and the top ten KEGG pathways to investigate their potential associations. Using MCC scores for filtering, the five most significantly associated diseases were identified as inflammation, infections, neoplasms, malnutrition, and vitamin A deficiency (Figure 5B). This predictive analysis suggests that these metabolites could be associated with these conditions in silico, but no efficacy is claimed without experimental validation. Among the enriched KEGG terms, the top three most significant were pathways in cancer (hsa05200), the thyroid hormone signaling pathway (hsa04919), and the estrogen signaling pathway (hsa04915). We pinpointed five key functional nutritional metabolites: retinol (vitamin A1), cis-5-dodecenoic acid, glucopyranose, N-acetyl-L-tryptophan, and D-arabinose. Five core targets were also identified: ESR2, MAPK1, MAPK3, PRKCA, and PPARG.

### 3.6. Molecular Docking Analysis

**Figure 6:**
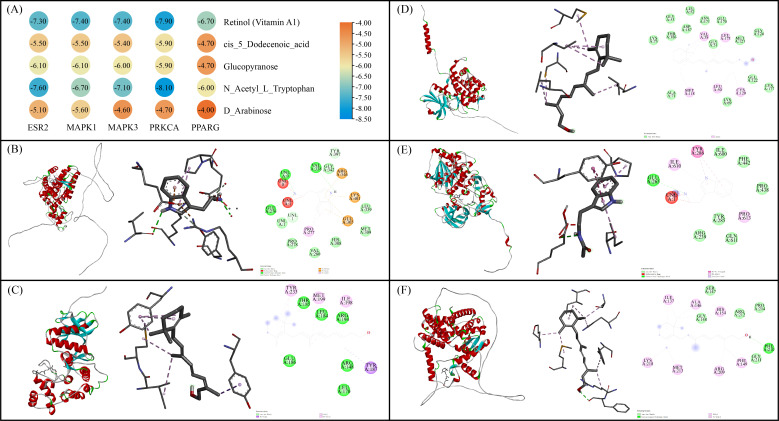
Molecular docking models illustrating the interactions between bioactive nutritional metabolites and their prioritized protein targets within *Ocimum*. (**A**) Heatmap of binding energy. (**B**) ESR2 with N-Acetyl-L-Tryptophan. (**C**) MAPK1 with Retinol (Vitamin A1). (**D**) MAPK3 with Retinol (Vitamin A1). (**E**) PRKCA with N-Acetyl-L-Tryptophan. (**F**) PPARG with Retinol (Vitamin A1).

Molecular docking of the five core targets and five candidate core metabolites was conducted as a predictive tool, without positive controls or re-docking. The binding energies are presented only for relative comparison and are not evidence of biological activity (Figure 6A). All calculated binding energies were negative, which is a prerequisite for in silico binding. However, these values are model-dependent and lack experimental context. They are used only to rank relative potential, not to infer biological stability or activity. Detailed analysis revealed the following: The binding energy of ESR2 and N-Acetyl-L-Tryptophan was the lowest at −7.60 kcal/mol, indicating the highest stability. The binding interfaces were held together by a wide range of forces, including conventional hydrogen bonds and carbon–hydrogen bonds, van der Waals contacts, and some “unfavorable bumps” and various pi-effects (pi–cation, pi–anion and pi–alkyl) (Figure 6B). The binding energy of MAPK1 and Retinol (Vitamin A1) was −7.40 kcal/mol, which was the lowest in their group, suggesting high stability. Van der Waals forces along with pi–sigma, alkyl and pi–alkyl (Figure 6C) interactions were included. In a similar manner, MAPK3 and Retinol (Vitamin A1) showed the least binding energy value of −7.40 kcal/mol, which corresponds to the most stable compound. They interacted mostly by van der Waals and alkyl (Figure 6D). In spite of unfavorable bumps, the molecular complex was held by covalent linkages, pi–alkyl and pi–pi T-shaped interactions, conventional hydrogen bonds and van der Waals contacts. The model with Retinol (Vitamin A1) bound to PPARG showed the highest stability (−6.70 kcal/mol), with the main contributors being alkyl/pi–alkyl interactions, conventional hydrogen bonds, and van der Waals interactions (Figure 6F). These results provide insights into the principal types of interaction between the functional nutritional metabolites from *O. basilicum* and their receptor targets, where the van der Waals force is found to be the main force in keeping the ligand receptor stable. Of particular interest in this predictive analysis are Retinol (Vitamin A1) and N-Acetyl-L-Tryptophan, which were identified as the most promising candidates and need to be further validated in vitro or in vivo.

### 3.7. Metabolic Pathway Analysis

**Figure 7:**
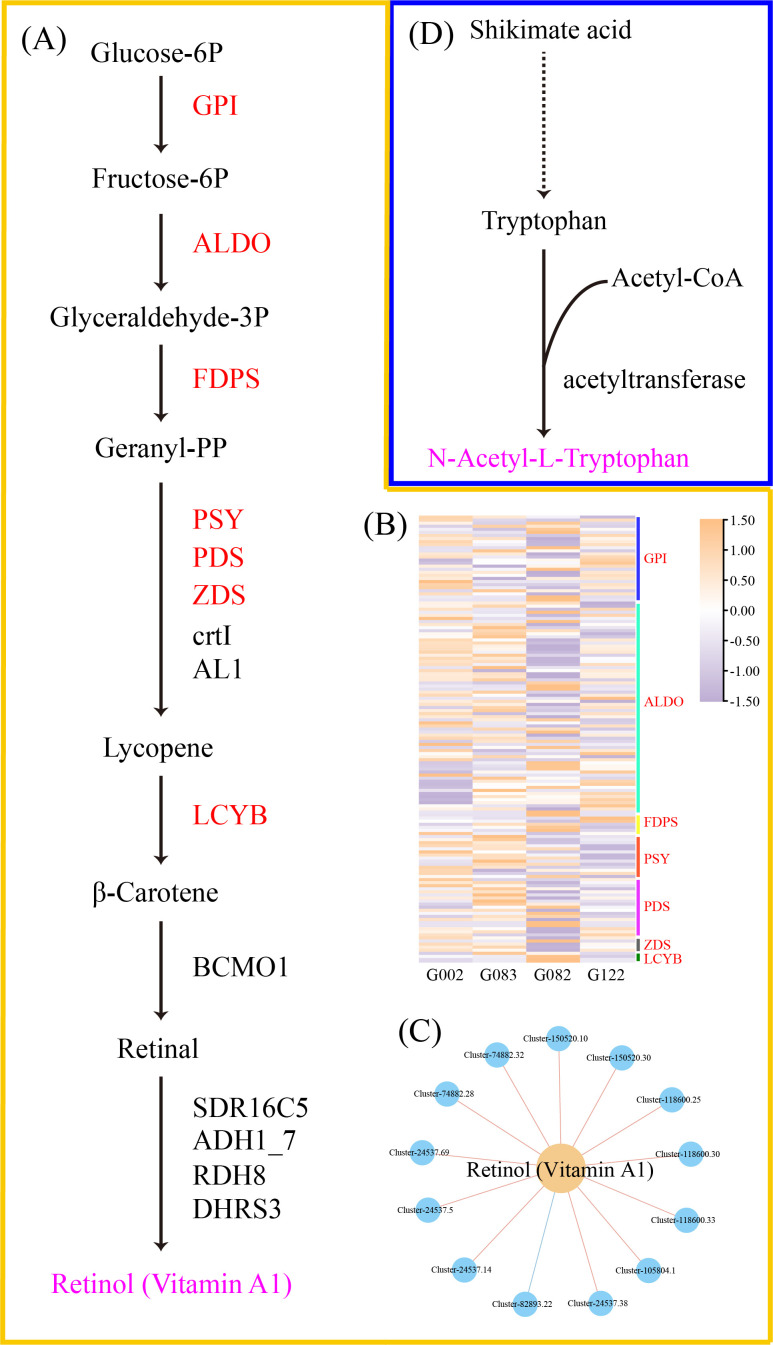
Metabolic pathway analysis of *Ocimum basilicum* N-Acetyl-L-Tryptophan and Retinol (Vitamin A1). (**A**) Retinol (Vitamin A1) metabolic pathway analysis. (**B**) Heatmap of differentially expressed genes. (**C**) Regulatory network of Retinol (Vitamin A1) with candidate differentially expressed genes. (**D**) N-Acetyl-L-Tryptophan metabolic pathway analysis.

In our study, we analyzed the metabolic pathways of the two core functional nutritional metabolites identified. For Retinol (Vitamin A1), we found that Glucose-6P serves as the initial substrate. This metabolite is first converted to Fructose-6P by the action of GPI (glucose-6-phosphate isomerase) enzymes, then further transformed into Glyceraldehyde-3P by ALDO (fructose-bisphosphate aldolase) enzymes. Subsequently, FDPS (farnesyl diphosphate synthase) enzymes catalyze the conversion of Glyceraldehyde-3P into Geranyl-PP. The biosynthesis of lycopene from geranyl-PP requires a sequence of enzymatic steps mediated by 15-cis-phytoene synthase (PSY) and a suite of desaturases, specifically zeta-carotene desaturase (ZDS) and multiple phytoene desaturases (PDS, crtI, and AL1). LCYB (lycopene beta-cyclase) enzymes then catalyze the formation of β-Carotene from Lycopene, which is further converted to Retinal by BCMO1 (beta-carotene 15,15′-dioxygenase) enzymes. Finally, Retinal is transformed into Retinol (Vitamin A1) through the action of multiple enzymes (Figure 7A).

Using transcriptomic data, we identified 145 differentially expressed genes related to this metabolic pathway. These genes include 28 GPI, 69 ALDO, 7 FDPS, 14 PSY, 19 PDS, 5 ZDS, and 3 LCYB genes. These genes exhibit diverse expression patterns in the four basil materials studied (Figure 7B).

To identify candidate genes whose expression is correlated with Retinol (Vitamin A1) content, we constructed a regulatory network between Retinol (Vitamin A1) and the candidate genes. We found 13 candidate genes that showed a high correlation coefficient (>0.95, *p* < 0.05) with Retinol (Vitamin A1) based on only four accessions. Such high correlation values are statistically fragile with this sample size and should not be interpreted as causal or robust without independent replication (Figure 7C). These genes include 1 GPI (Cluster-82893.22), 6 ALDO (Cluster-24537.14, Cluster-24537.38, Cluster-24537.5, Cluster-24537.69, Cluster-74882.28, and Cluster-74882.32), 2 PSY (Cluster-150520.10 and Cluster-150520.30), 3 PDS (Cluster-118600.25, Cluster-118600.30 and Cluster-118600.33), and 1 LCYB (Cluster-105804.1).

For N-Acetyl-L-Tryptophan, the initial substrate is Shikimate acid. Through a series of enzymatic reactions, it is converted to Tryptophan. Then, in the presence of Acetyl-CoA and under the action of Acetyltransferase, it is transformed into N-Acetyl-L-Tryptophan (Figure 7D).

### 3.8. qPCR-Based Verification of Transcriptomic Datasets

To corroborate the transcriptomic profiles obtained from RNA sequencing, we performed quantitative real-time PCR (qPCR) on 12 specific candidate genes across four materials (G002, G083, G082, and G122). The expression levels measured by qPCR were compared with the corresponding FPKM values from the transcriptome analysis.

**Figure 8:**
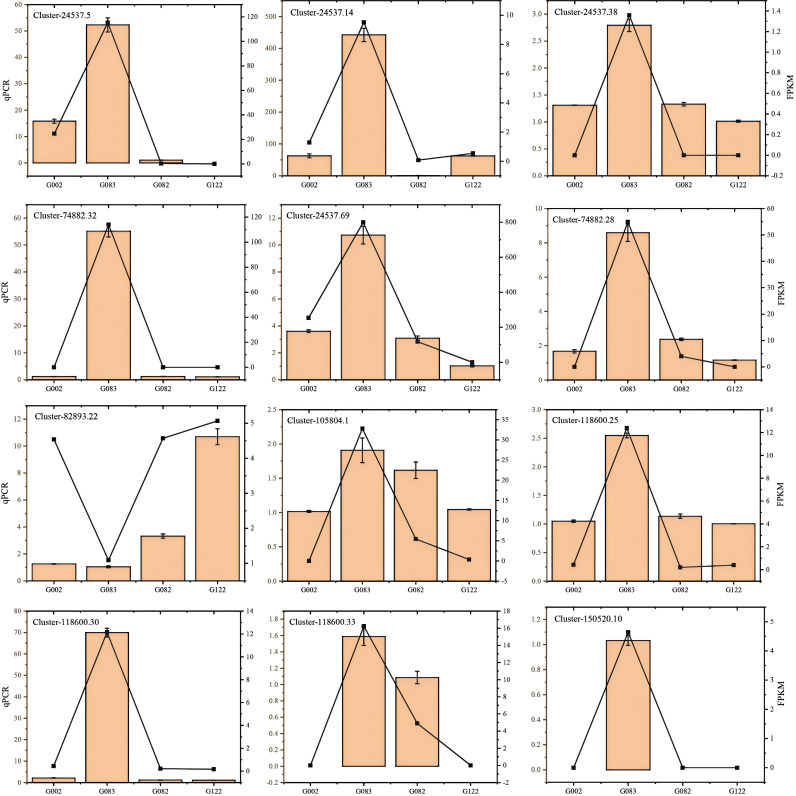
Expression analysis of candidate genes by qPCR. Bars represent the relative expression levels determined by qPCR, and the line graph shows the corresponding FPKM values from RNA-seq.

Overall, the qPCR results showed a consistent trend with the RNA-Seq data for the majority of the tested genes (Figure 8). For instance, genes such as Cluster-24537.14 (ALDO) and Cluster-24537.38 (ALDO) exhibited higher expression levels in material G083, which corresponded with elevated FPKM values in the transcriptome data. Similarly, Cluster-24537.5 (ALDO) and Cluster-74882.32 (ALDO) displayed markedly higher expression in G083 compared to other materials, aligning well with the FPKM trends.

However, some discrepancies were observed, particularly in cases where FPKM values were reported as zero while low but detectable expression was recorded by qPCR. For example, Cluster-24537.38 (ALDO) showed negligible FPKM (0) in G002 and G082, yet low levels of expression were detected via qPCR. Similar observations were made for Cluster-74882.28 (ALDO) and Cluster-74882.32 (ALDO). These differences likely reflect the higher sensitivity of qPCR in detecting low-abundance transcripts, which may fall below the detection threshold of RNA-Seq.

Notably, for genes such as Cluster-24537.69 (ALDO) and Cluster-118600.30 (PDS), both methods consistently captured significant upregulation in G083, further supporting the reliability of the transcriptome data. Conversely, genes including Cluster-150520.10 (crtB) and Cluster-118600.33 (PDS) showed minimal expression across all materials in both datasets.

The correlation between qPCR and FPKM values confirms that the transcriptome data broadly reflects actual gene expression patterns. Discrepancies in low-expression genes highlight methodological differences in detection limits between the two platforms, yet do not undermine the overall consistency of the expression trends.

## 4. Discussion

With the growing interest in health and natural products, *Ocimum basilicum*, being a nutraceutical plant, will draw increasing attention for its nutritional and medicinal benefits. In the food sector, *O. basilicum* holds potential for developing various functional foods, such as nutrient-dense supplements and leisure foods with unique flavors and health-promoting properties [36]. Clinically, exploring the bioactive compounds in *O. basilicum* could pave the way for novel therapeutic agents or health products aimed at preventing and treating a range of diseases [37].

Practically, the multi-omics data set obtained in this study can be useful for breeding programs focusing on nutritional quality. In particular, accessions G083 (high retinol) and G082 (high N-acetyl-L-tryptophan) could be used as parent materials for the development of functional food ingredients or nutraceutical extracts (if further validated).

Nutritional metabolites in botanical systems have been the target of many studies due to their critical role in biological infrastructure and their importance to human nutrition. Comparative studies have been carried out on a wide range of species, and metabolic fingerprints have been identified, such as *Houttuynia cordata*, which has been found to contain 92 amino acids (and derivatives), 65 saccharides and 20 vitamins which all make significant contributions to the nutritional properties of the plant [38]. In the same way, the Six Coarse Cereals contained ninety three (93) amino acids related compounds, seventy two (72) lipids, twenty one (21) carbohydrates and twenty one (21) vitamins, which shows that coarse cereals are nutrient-rich foods [39]. Key metabolites such as isoflavones, organic acids, lipids, sugars and amino acids were characterized in soybeans, and isoflavones, free amino acids and fatty acids were the major discriminators between the wild and cultivated varieties [40]. Interestingly, the quality of walnuts was found to be strongly correlated with glycerophospholipid dynamics, highlighting lipid metabolism as an important factor to consider in the quality of nuts [41]. To systematically characterize the nutrient composition of *O. basilicum*, four accessions of *O. basilicum* were profiled by UPLC-MS/MS. The 443 nutritional metabolites identified were mostly lipids (191 species, 43.12%) and amino acid-related compounds (183 species, 41.31%), followed by saccharides (56, 12.64%) and vitamins (13, 2.93%). Principal component analysis (PCA) and differential metabolite screening showed that there was a significant difference in the compositions between the accessions, where each accession had a distinct metabolite profile. Interestingly, the amount of lipids (191) is similar to that of a wider investigation of six Ocimum accessions comprising of three different species *Ocimum × africanum*, *O. tenuiflorum* and *O. gratissimum* [13], which indicates a conserved lipid metabolic capacity in the genus. Qualitative differences in lipid classes and predominant species however, suggest that metabolic specialization is dependent on genotypes.

In addition to their basic nutritional functions, amino acids, vitamins, saccharides and lipids also have significant pharmacological activity. Moringa oleifera leaves, for example, which are rich in amino acids like glutamic acid and arginine, have been found to exert hypoglycemic and hypolipidemic effects by the dual inhibition of α-glucosidase and pancreatic lipase. These actions include lipid metabolism biomarker control (such as triglycerides, low-density lipoprotein cholesterol) and improving glucose uptake, demonstrating their therapeutic potential in metabolic disorders [42]. In the same way, the fruits of *Nitraria tangutorum* have an immune regulation function, which is manifested by the synergistic effect of vitamin C, vitamin E and polysaccharides on immune regulation, thereby attenuating neurasthenia and fatigue. Vitamins neutralize free radicals, thereby protecting against aging, and polysaccharides help to control gut microbiota in order to strengthen host defense mechanisms [43]. Polysaccharides extracted from fruiting bodies or mycelia of *Ganoderma* spp. are known to have strong effects on the immune system and antitumor effects. These polysaccharides activate both innate immunity (macrophages) and adaptive immunity (T lymphocytes); therefore, they offer good immune surveillance and the ability to destroy tumor cells [44]. Furthermore, fungal species such as *Cordyceps sinensis*, *Inonotus obliquus*, *Coprinus comatus*, *Phellinus linteus*, *Sparassis crispa*, *Agaricus bisporus*, *Agaricus subrufescens*, and *Pleurotus* spp. demonstrate significant hypoglycemic effects, underscoring their utility in glycemic management [45]. Omega-3 polyunsaturated fatty acids (PUFAs) (particularly DHA) have anti-inflammatory properties and reduce the release of pro-inflammatory cytokines (e.g., TNF-α); they also maintain neuronal synaptic plasticity, providing neuroprotective effects against inflammation [46]. This study, based on network pharmacology, revealed the intricate relationships between bioactive metabolites, potential targets, associated pathologies, and signaling pathways, offering a holistic perspective on the therapeutic potential of *O. basilicum*. Further functional annotations using GO and KEGG databases explained the beneficial effect of these botanical nutrients against neoplastic growth, inflammatory responses, infectious agents and nutritional deficiencies (vitamin A deficiency).

Molecular docking is an advanced computational approach for the simulation of the dynamic interaction of small molecules with biological receptors, which enables a valid estimation of the small molecules binding conformation and thermodynamic affinity [47]. The docking simulations in this work showed that retinol (vitamin A1) and N-acetyl-L-tryptophan are strong binders for five key therapeutic targets, lending to their proposed role in intervening in diseases and mechanistic insights for drug discovery. *Ocimum* produces a variety of vitamins such as vitamin A, C, E, K and B-complex vitamins [48,49]. In plants, vitamin A is involved in controlling both photomorphogenesis and leaf morphogenesis, and in *Ocimum*, it affects the photosynthetic efficiency by keeping the chloroplasts intact. In regards to human health, vitamin A is essential to the synthesis of rhodopsin in the photoreceptors in the retina, and a deficiency of vitamin A is known to be associated with night blindness and defective epithelial barrier function [50]. Interestingly, an acetylated derivative of L-tryptophan, N-acetyl-L-tryptophan, was found to be a pharmacologically relevant *O. basilicum* metabolite with multimodal bioactivity (neuroprotection, anti-inflammation, antioxidant and metabolic modulation). The mechanism enhances the endogenous antioxidant defense mechanisms through the upregulation of glutathione (GSH), thereby scavenging the reactive oxygen species (ROS)-mediated oxidative stress. Interestingly, accession G082 showed a higher accumulation of N-acetyl-L-tryptophan compared to other accessions, which means that it could be a lead compound for therapy. In addition, one bioactive form (retinol, vitamin A1) present only in the accession G083 of *O. basilicum* is highlighted as a target for nutraceutical development.

Comparative transcriptomic analyses of four accessions of *O. basilicum* were performed to gain insights into the molecular basis of the differential accumulation of the functionally critical metabolites. This strategy helped to define retinol’s (vitamin A1) biosynthetic pathway and identify differentially expressed genes (DEGs) that control the retinol metabolic pathway. Retinol accumulation dynamics and transcriptomic signatures integrated into an analysis identified a co-expression network that involved GPI, ALDO, PSY, PDS and LCYB as key genes involved in retinol biosynthesis. Transgene functional validation showed that the co-expression of codon-optimized sZmPSY and sPaCrtI led to an increase in the level of β-carotene in the endosperm and, consequently, an increase in the level of vitamin A1 [51].

We acknowledge several important limitations of this study. The research only involved four *Ocimum basilicum* accessions. Given the many *Ocimum* species with possible differences in nutrition, metabolites, and medicinal properties, future studies examining more varieties could identify valuable resources. Additionally, all plant materials were grown under standardized nursery conditions. While this controlled setting minimizes environmental noise and ensures reproducibility, it does not fully reflect the complex agronomic and ecological factors present in open-field cultivation. Whether the observed metabolite profiles remain stable under field conditions requires further investigation. Regarding research depth, although network pharmacology and molecular docking can predict key metabolite targets and mechanisms, these results are purely predictive and lack in vitro or in vivo validation; no positive controls or re-docking were performed, and binding energies are presented only for relative comparison. Transcriptome sequencing reveals key metabolite pathways and gene expression changes, but gene function and regulation research is insufficient. Potential batch effects inherent to multi-omics profiling cannot be completely ruled out, despite standardized sample processing and data acquisition. Future studies should incorporate randomized experimental designs and more rigorous quality controls to mitigate this issue. Importantly, correlations between gene expression and metabolite content (*r* > 0.95 for retinol) are based on only four accessions, making them statistically fragile; high correlation coefficients are not evidence of causality and must be interpreted with extreme caution. To address these shortcomings, one could use gene-editing technologies like CRISPR/Cas9 to edit key genes and study their effects on metabolite synthesis and pathways, which would clarify gene functions and regulation.

Recent studies have highlighted the complex interplay between hormonal signals and molecular pathways that govern secondary metabolism in plants, which aligns with the transcriptomic analysis of biosynthetic genes and regulatory insights for key metabolites in this study [52]. An additional integrative step was taken to link the three omics layers. Metabolomics identified the differential accumulation of retinol and N-acetyl-L-tryptophan across accessions. Transcriptomics revealed candidate biosynthetic genes (*PSY*, *LCYB*, *ALDO*) whose expression patterns correlated with metabolite levels, and network pharmacology predicted potential protein targets (ESR2, MAPK1) for these metabolites. By mapping these three layers onto the same biological context (vitamin A metabolism and inflammatory pathways), we provide a multi-level, hypothesis-generating framework. However, direct causal links between gene expression, metabolite abundance, and pharmacological activity remain unvalidated, and future experiments (gene knockout or heterologous expression) are needed to test these connections. From a nutritional and functional perspective, the health-promoting potential of plant metabolites, including vitamins, amino acids, and their derivatives, is increasingly supported by evidence linking them to metabolic and inflammatory pathways [53]. Consistent with this, our predictive analysis suggests that vitamin A1 and N-acetyl-L-tryptophan may interact with targets involved in cancer and estrogen signaling, providing a hypothesis-generating foundation for future nutraceutical exploration.

## 5. Conclusions

In this study, an integrative multi-omics strategy was applied to comprehensively characterize the nutritional metabolite composition and associated regulatory mechanisms of *Ocimum basilicum*. A diverse array of nutritional metabolites was identified, among which retinol (Vitamin A1) and N-acetyl-L-tryptophan emerged as key constituents with potential functional relevance. By integrating transcriptomics, metabolomics, molecular docking, and network pharmacology analyses, this work predictively explored the biosynthetic basis and in silico molecular interactions of these metabolites, which remain to be experimentally confirmed. Importantly, qPCR validation further corroborated the RNA-Seq results, indicating that the transcriptomic analysis reliably captured overall gene expression trends. Collectively, these findings provide a robust multi-omics dataset and mechanistic insights that support hypothesis generation for the rational utilization of *O. basilicum* in nutritional research pending experimental validation and offer a foundational reference for future studies on plant metabolic regulation and functional metabolite discovery.

## Figures and Tables

**Table 1:** Primers for quantitative real-time PCR.

Gene Name	Forward (5′~3′)	Reverse (5′~3′)
GAPDH	AACATTATCCCCAGCAGCAC	TAGGAACTCGGAATGCCATC
Cluster-82893.22	GCATCGCATGCTCGTTTTCT	GACGGTCTGATTGTGGAGCA
Cluster-24537.14	GTGCCGAATGCAAGGACAAG	TCGAGATTGAGCTGAAGCCAT
Cluster-24537.38	TGAGCATTCCCAATGGTCCC	GGTGTGACCATGCTAGGCTT
Cluster-24537.5	CTCAGCGTCACCCCTCAAAT	GCAACAGTTTTCGCGGTCTT
Cluster-24537.69	CGAATCAAACGCTACCTGCG	CGACTTTGATTCCGGGGACA
Cluster-74882.28	AGCATCCAGCAGAATGCACA	AGTCCCCTCCAGTAGCACAT
Cluster-74882.32	GCCCGAGATCCTGACAGATG	ATACGATCCCTGGTACCGCT
Cluster-150520.10	GCTGCATTGGCCTTAGGGAT	TTCACCCCATTCTCCGCATC
Cluster-118600.25	ACAAGCCAGGAGAGTTCAGC	ACAAGCCAGGAGAGTTCAGC
Cluster-118600.30	GGAGCTGGTTTTTGCACCTG	AAGGTTCGGTGCCAGGTATG
Cluster-118600.33	CGAAGCTATTCCCAGACGAGA	CCCTGACAGAACTGCACCTT
Cluster-105804.1	GCCGGTTTTGCCTCAAAGAG	AGAAGAACTCCCGTTGTCGC

## Data Availability

The raw RNA-Seq datasets resolved in this investigation are publicly accessible through the NCBI Sequence Read Archive (SRA) depository under the explicit accession number PRJNA1344911, adhering fully to the framework of the International Nucleotide Sequence Database Collaboration (INSDC).
